# Temporality of change in Ki67 labelling following neoadjuvant systemic chemotherapy of breast cancer—the common drop often subsides with time

**DOI:** 10.1007/s00428-025-04263-7

**Published:** 2025-11-13

**Authors:** Ádám Ferenczi, Tamás Lantos, Gábor Cserni

**Affiliations:** 1 Department of Pathology, Albert Szent-Györgyi Medical School, University of Szeged, Állomás U.1, 6725 Szeged, Hungary; 2 Department of Medical Physics and Informatics, Albert Szent-Györgyi Medical School, University of Szeged, Korányi Fasor 9, 6720 Szeged, Hungary; 3 Department of Pathology, Nyíri Út 38, Bács-Kiskun County Teaching Hospital, 6000 Kecskemét, Hungary

**Keywords:** Ki67, Breast cancer, Temporality, Neoadjuvant chemotherapy, Post-neoadjuvant

## Abstract

Ki67 immunohistochemistry is widely used in clinical settings to assess tumour proliferation in breast cancer, yet the temporal changes in Ki67 labelling index following neoadjuvant chemotherapy (NACT) are underexplored. Matched core biopsy and surgical excision specimens of invasive breast cancers were collected, and the Ki67 immunostained slides were examined. Ki67 labelling index was determined and statistically evaluated based on the time elapsed between the last cycle of NACT and surgery. Subgroup analyses were carried out based on surrogate molecular subtypes, Residual Cancer Burden (RCB), and European Working Group for Breast Screening Pathology tumour regression (TR) categories. The study included 54 paired breast cancer cases post-NACT. Most tumours were RCB-II (*n* = 39) and TR2b (*n* = 20). The average time from NACT completion to surgery was 40.3 days. Of three methods (including image-analysis and sample-counting), eye-balling estimation provided the best linear regression fit (*p* = 0.003, *R*^2^ = 0.1548). Subgroup analysis showed reduced proliferation suppression over time in HER2-positive, triple-negative, and luminal B-like tumours, as well as RCB-II, RCB-III, TR2b, TR2c, and TR3 tumour regression categories. Conversely, luminal A-like tumours, RCB-I, and TR2a categories showed an opposite trend, though limited by small sample size. Multiple studies highlight the prognostic value of Ki67 labelling index post-NACT, with pre- and post-treatment values and change between them serving as independent prognosticators, but our data suggest that the latter two may be time-dependent after the completion of NACT. The proliferation-reducing effect of NACT diminishes over time, particularly in non-luminal A-like tumours and those with poorer treatment responses.

**Supplementary Information:**

The online version contains supplementary material available at 10.1007/s00428-025-04263-7.

## Introduction

Ki67 is a nuclear protein expressed during the G1, S, G2, and M phases of cell division, but not in the resting state (G0); thus, its demonstration by immunohistochemistry (IHC) has been widely used to reflect tumour proliferation in breast cancer [1]. However, Ki67 also raises the problem of interobserver and intraobserver variability and suboptimal reproducibility [2–4].

The prognostic value of the Ki67 labelling index (LI) in breast cancer has been widely studied and a meta-analysis found preoperative (CNB-Ki67) and postneoadjuvant postoperative Ki67 LI (yKi67), as well as the change in Ki67 values from biopsy to postneoadjuvant surgical specimen (ΔKi67) to be independent prognosticators for both overall survival (OS) and recurrence-free survival (RFS) [5]. More precisely, prior research has found CNB-Ki67 LI to be a marker of worse prognosis and also a strong predictor of pathological complete response (pCR) and higher values forecasting a better response to chemotherapy [6–12]. For yKi67, higher values carry a worse prognosis and mark an increased risk of early local and distant relapse [5, 11, 13–15].

Despite both CNB-Ki67 and yKi67 being well documented as prognostic factors in breast cancer by multiple authors, the trend of change in Ki67 over time remains underexamined and less documented. In this study, we aimed to examine the temporality of changes in Ki67 LI values from core biopsies (CNB) to surgical excision specimens following neoadjuvant chemotherapy (EXC) in invasive breast cancers.

## Materials and methods

Invasive breast cancers treated with primary systemic chemotherapy with both pretreatment CNB and posttreatment EXC specimens diagnosed at the Bács-Kiskun County Teaching Hospital between 2009 and 2024 were selected. To be included in the study, the cases had to have both CNB and EXC specimens available with an assessable amount of both primary and residual invasive breast carcinoma to determine the grade (at least 2 mm^2^ area of the tumour), and Ki67 IHC. Cases with technically suboptimal staining, cut-out material, and only megaslides available with residual tumours were excluded, as well as cases from patients who also received endocrine treatment following the termination of chemotherapy and before surgery. For the remaining cases, the following data were extracted from the histology reports and patients’ digital charts: age, tumour type and grade, oestrogen and progesterone receptor (ER, PR) status (+ : positive or -: negative), human epidermal growth factor receptor-2 (HER2) status, Ki67 IHC results as original, eye-balling based estimation of the percentage of stained tumour cells (EB-EST), scores for tubule formation, nuclear pleomorphism and mitotic count according to the Nottingham grading system [16], mitotic index (MI: number of mitoses per 10 high power fields corresponding to 2 mm^2^), furthermore exact dates of surgery and the last cycle of neoadjuvant chemotherapy (NACT) before surgery, type of neoadjuvant treatment and response to neoadjuvant therapy. Grade and proliferation-related data were obtained for both the CNB and EXC specimens. For the EB-EST method, not only a guess of stained tumour cell percentage was used, but most of the time, areas of roughly 100 cells were virtually evaluated for the proportion of Ki67 positive cells, and the estimation was made using the distribution of such areas and their labelling proportions.

Besides the relatively subjective estimation, the proportion of Ki67-stained tumour cells was also determined using 2 more objective methods. Corresponding slides were collected from the archives and scanned for each case with a Pannoramic 250 scanner (3DHistech, Budapest, Hungary). Three images were taken from each slide with the SlideViewer software (3DHistech, Budapest, Hungary) at ×30 magnification, aiming to represent the tumour as well as possible; in cases with homogenous staining density, 3 random areas were photographed; for cases with heterogeneous Ki67 expression, we aimed to take 2 images reflecting the dominant density and one representing the minority, to allow some weighting for the average. We paid attention not to include pictures containing in situ (DCIS) components. These digital images were then the basis of further analyses.

One method (IR) used the ImmunoRatio 1.0c plugin [17] with the Fiji imagej.net image processing package, which determines positive and negative nuclei by analysing the image according to brown (immunostained, i.e., positive) and blue (haematoxylin stained, i.e., negative) colour thresholds. The source image scale (4.0 pixel(s)/µm) and correction equation (*y* = ax^3 + bx^2 + cx + d; *a* = 6.442, *b* = 0, *c* = 0.611, *d* = 0.43) values remained as default. The brown and blue colour thresholds were modified at each image to best represent the unique nuclear staining observable on each slide. A limitation of the software observed during analysis was the fact that in cases where the slide was understained or heavily overstained, or the cell density was remarkably high, the software had trouble differentiating single nuclei. This led to a blurring effect, which was unresolvable even with colour threshold adjustments because a decrease in colour sensitivity led to multiple nuclei not being recognised. In these troublesome cases, the colour thresholds were adjusted so that the results best represented all tumour cells. The minimal sensitivity was determined for each digital image to allow all tumour cell nuclei to be recognised, and the blurring effect was minimised to avoid false positive recognition. This limitation was not present on slides where the contrast between nuclear and cytoplasmic or background staining was more intense. During the analysis of slides that contained large foci of connective tissue or inflammatory cell infiltrate, the image was masked to only contain tumour cells, thus removing all other nuclei from the recognition analysis.

The third method of Ki67 quantification was a grid counting (GRID) following the description of Vörös et al. [18]: all images were exported to Microsoft PowerPoint, and a grid of horizontal Lines 1.5 cm apart, starting from the middle of the slide in both directions, was spread over the images. The nuclei which were intersected by these lines were then counted manually, and the ratio of positive to all nuclei was calculated.

To characterise the cases, the Ki67 staining percentages were used. For the EB-EST method, the whole slide and the whole available material were considered for pretreatment CNBs, and a single tumour slide was Ki67 stained as representing the posttreatment EXC specimen; the whole slide examination was the basis for the estimation of the average labelling. For the IR and GRID methods, the average of the quantification of the 3 representative images was taken. By the end, for each tumour, there were three pairs of CNB and EXC Ki67 labelling values, to follow the time-dependency of changes in proliferation after the last cycle of NACT. Data were collected and managed in Microsoft Excel.

Cases were grouped for subset analyses. For molecular subtypes, we used an IHC-based approach distinguishing between HER2-positive (scored either 3 + or being amplified on in situ hybridisation [19]), triple-negative (TN: ER-PR-HER2-), and ER+HER2-, with a 1% staining required for ER+ or PR+ [20]. One single case of ER-PR+HER2- was lumped with the ER+HER2- cases. The response was categorised according to the Residual Cancer Burden (RCB) main classes I to III [21] which also includes parameters of the lymph nodes after NACT, and alternately, according to tumour response (TR) categories proposed by the European Working Group for Breast Screening Pathology: TR2a with < 10% residual cancer, TR2b with 10 to 50% residual cancer, TR2c with > 50% residual cancer but signs of regression, and TR3 for cases with no regression [22]. Examples of pre- and post-NACT cases with different TR categories are shown in Supplementary Fig. 1.

Linear regression analyses were performed to evaluate the effect of the time elapsed between the last cycle of chemotherapy and surgery (independent variable) on the change between starting and end values (i.e., the difference between EXC and CNB values, in this order; dependent variable). The model fits were quantified by the R-squared statistic (coefficient of determination; *R*^2^). Subgroup analyses were carried out by surrogate molecular subtypes, RCB, and EWGBSP regression categories, respectively.

Agreement between two measurement methods was assessed using Bland–Altman analysis. For each paired observation, the difference between the two methods was plotted against their mean. The mean difference (bias) and the 95% limits of agreement (mean difference ± 1.96 × standard deviation of the differences) were calculated to quantify the agreement. A linear regression of the differences in the mean values was performed to check for proportional bias. All statistical analyses were conducted using R (version 4.4.1). *p*-values of less than 0.05 were considered to be statistically significant.

## Results

After all exclusions, a total of 54 paired cases were included in our study. Clinical data of the patients and tumours are summarized in Supplementary Table 1. The most common surrogate molecular subtype observed was luminal B-like (*n* = 22), followed by TN (*n* = 16), HER2-positive (*n* = 12), and finally luminal A-like (*n* = 4). The majority of the tumours showed regression grades of RCB-II (*n* = 39) and TR2b (*n* = 20). The average time between the last cycle of NACT and surgery was 40.3 days (range: 8–82).

PST in 52/54 cases consisted of a taxane-containing regimen, most often involving the sequential administration of epirubicin and docetaxel, but some patients received a taxane plus platinum regimen or other taxane-containing regimens. As for the two remaining cases, one consisted of 3 cycles of epirubicin (as the patient declined further preoperative chemotherapy), and the other consisted of capecitabine. Ten out of 12 patients with HER2-positive tumours also received anti-HER2 treatment in the form of trastuzumab (5 ER+ and 1 ER- tumours) or a dual blockade with trastuzumab and pertuzumab (4 ER- tumours).

**Fig. 1:**
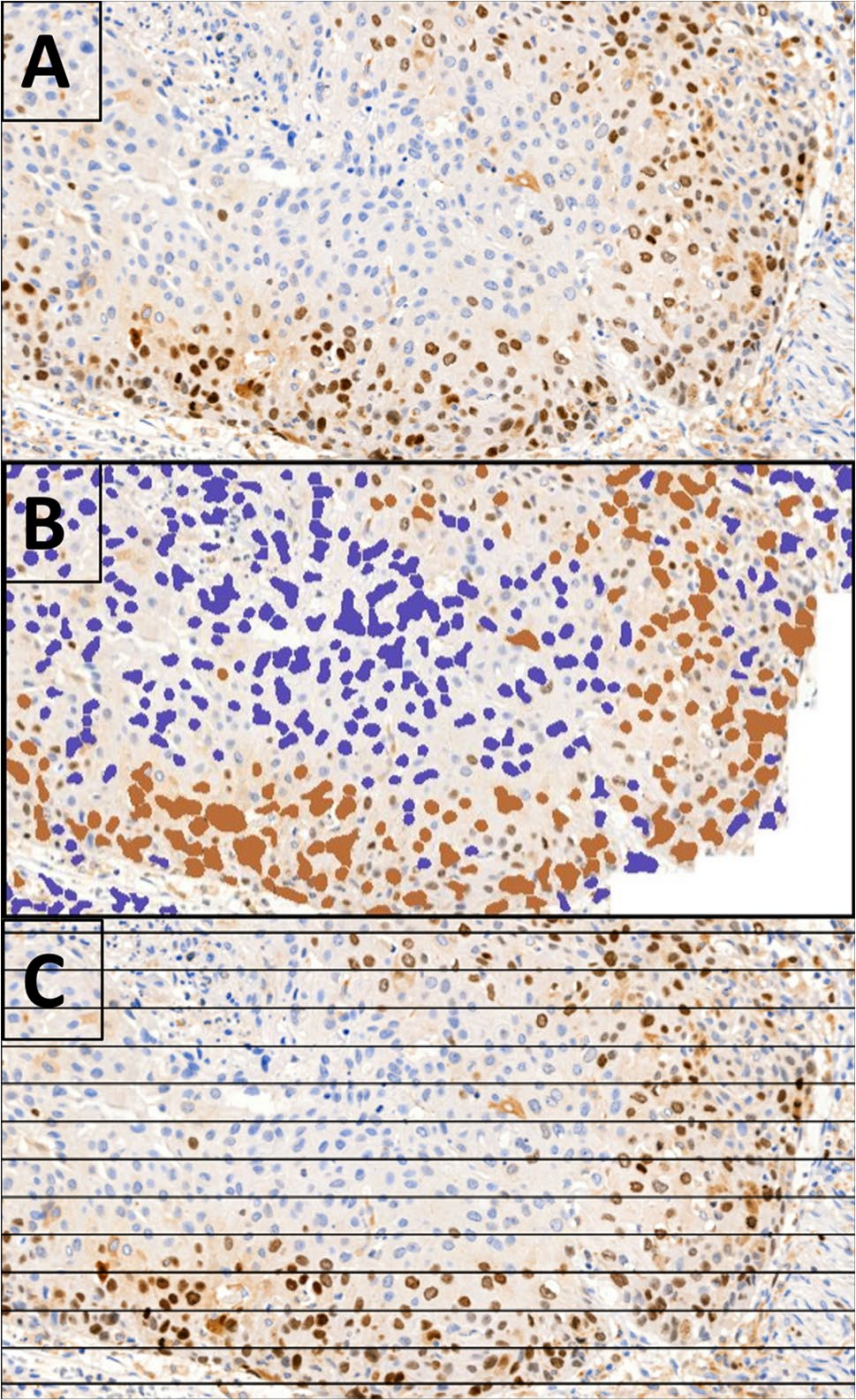
Illustration of the methods used (Ki67, 30x). **A** Eye-balling estimation (EB-EST) was made after examining the whole slide, looking at areas of about 100 cells and the number of labelled nuclei, and trying to average these; **B** ImmunoRatio (IR); relatively good contrast between the blue and brown colours helps the software accurately analyse the scanned Ki67 stained slide image; and **C** the GRID method utilized gridlines spaced equally on top of the scanned slide image and the intersected nuclei were counted

All cases were reported by the same pathologist, or the slides reported by others were reviewed by the same pathologist (GC), to limit interobserver variation. The same microscope was used for scoring mitoses during the period. A representation of the results of the IR method, as well as a representation of the gridlines used for the GRID method, is illustrated in Fig. 1.

**Fig. 2:**
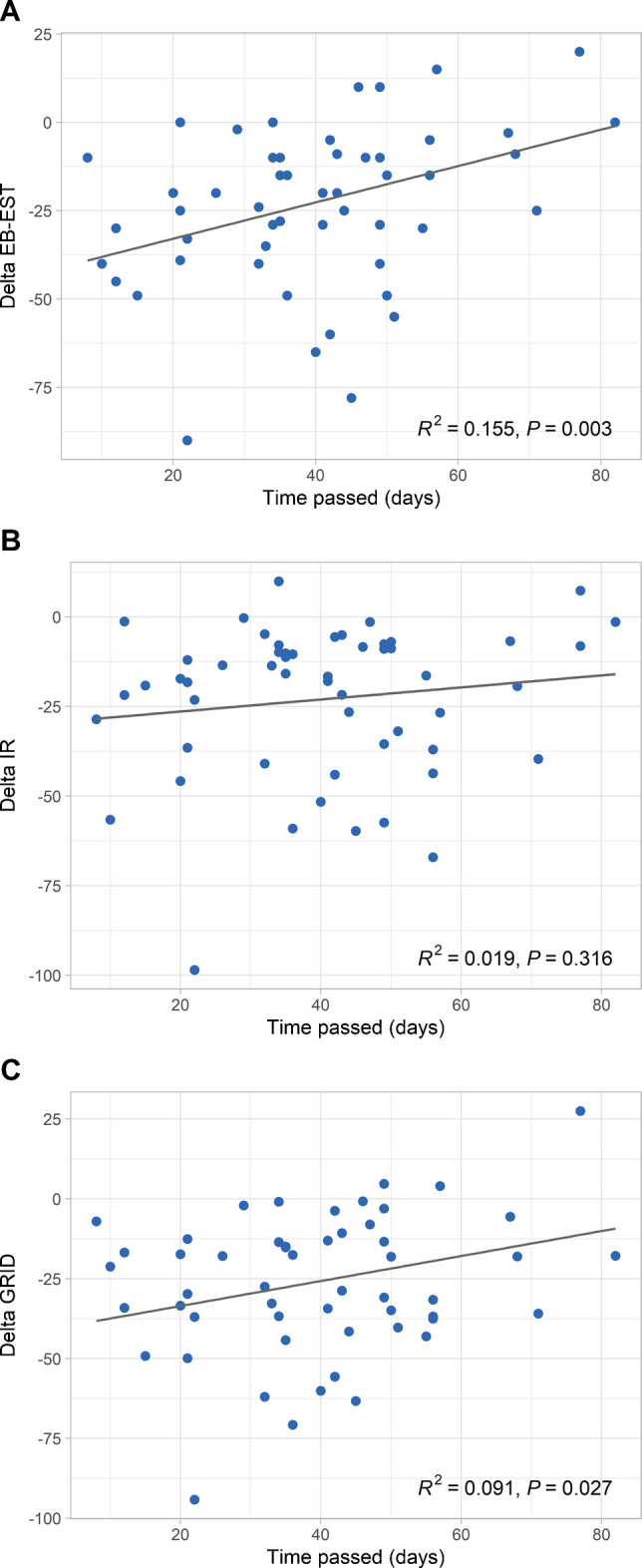
Scatter plot (each point represents an observation) with the fitted trend (line) and model fit (R-squared) illustrating the relationship between change (delta) in the value of Ki67 labelling index and days passed between last cycle of systemic chemotherapy and surgery for each observation method included in the study such as **A** eye-balling based estimation (EB-EST), **B** ImmunoRatio image processing package (IR), and **C** cell counting with the help of gridlines spread over the scanned slide image (GRID)

Out of all methods, the best goodness of fit for the linear regression model was achieved by the EB-EST method, indicating an upward trend, thus a decreasing change in Ki67 LI over time when all cases were grouped (*p* = 0.003, *R*^2^ = 0.1548). The linear regression results and *R*^2^ values for each quantification method are presented in Fig. 2.

**Fig. 3:**
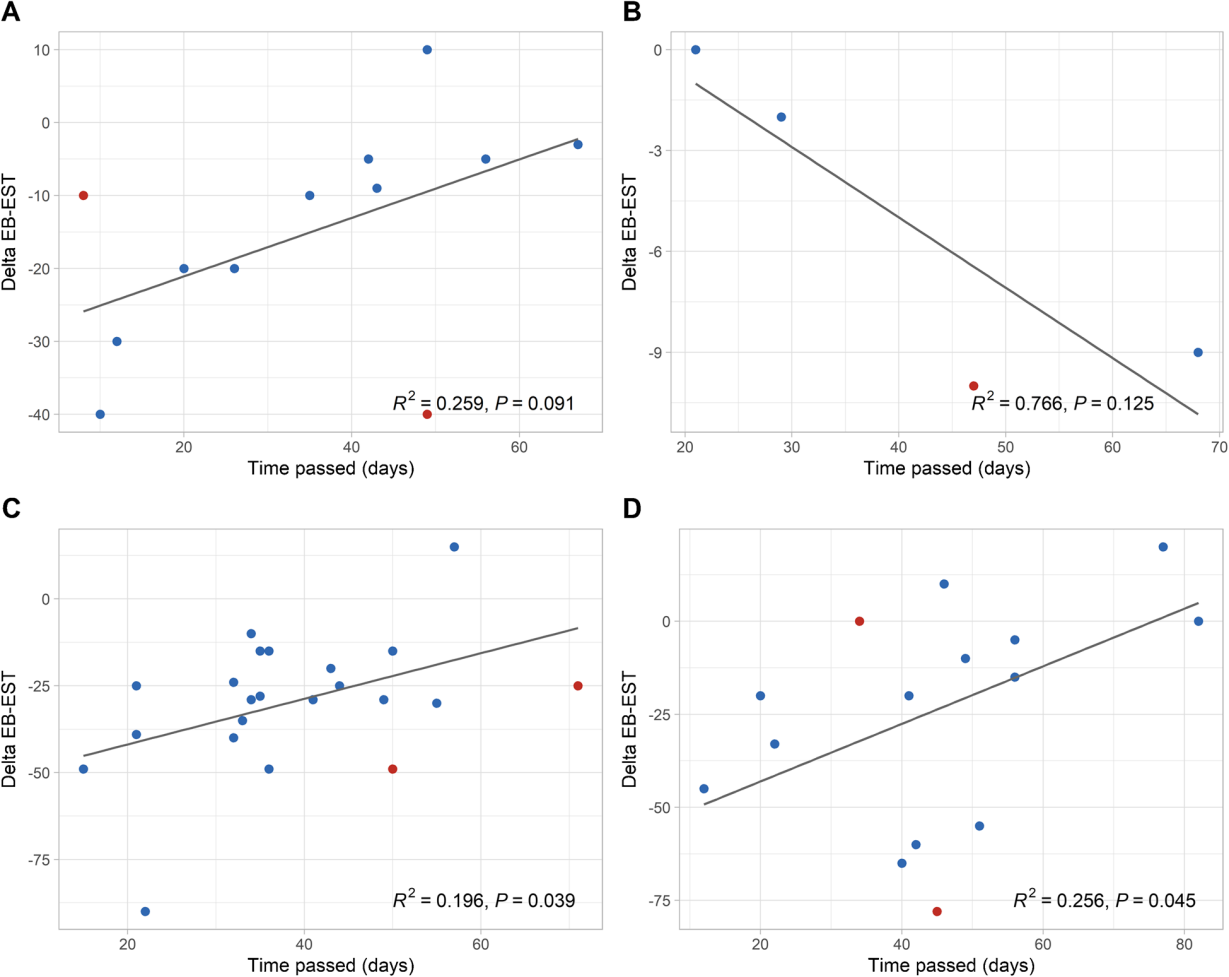
Scatter plot (each point represents an observation) with the fitted trend (line) and model fit (R-squared) illustrating the relationship between change (delta) in the value of Ki67 labelling index and days passed between last cycle of systemic chemotherapy and surgery for eye-balling based estimation (EB-EST) according to surrogate molecular subtypes (subgroup analysis) such as **A** HER2+, **B** luminal A-like, **C** luminal B-like, and **D** TN tumours. Statistical outliers are indicated in red. Footnote: HER2+, human epidermal growth factor receptor-2-positive breast cancers; TN, triple-negative breast cancers

In the subgroup analysis based on surrogate molecular subtype (Supplementary Table 2), luminal A-like tumours had the best fit with the linear regression model (*p* = 0.125, *R*^2^ = 0.766), indicating an increasing rate of proliferation blocking effect as time passes after the last cycle of NACT, albeit this subgroup contained the fewest number of cases (*n* = 4). HER2-positive (*p* = 0.091, *R*^2^ = 0.2589), TN (*p* = 0.045, *R*^2^ = 0.2563), and Luminal B-like (*p* = 0.039, *R*^2^ = 0.1961) tumours all demonstrated an upward trendline in the linear regression model, indicating that these tumours had a decrease in the proliferation blocking effect of NACT over time. The results of the subgroup analysis based on surrogate molecular subtypes are demonstrated in Fig. 3, alongside the *R*^2^ values.

**Fig. 4:**
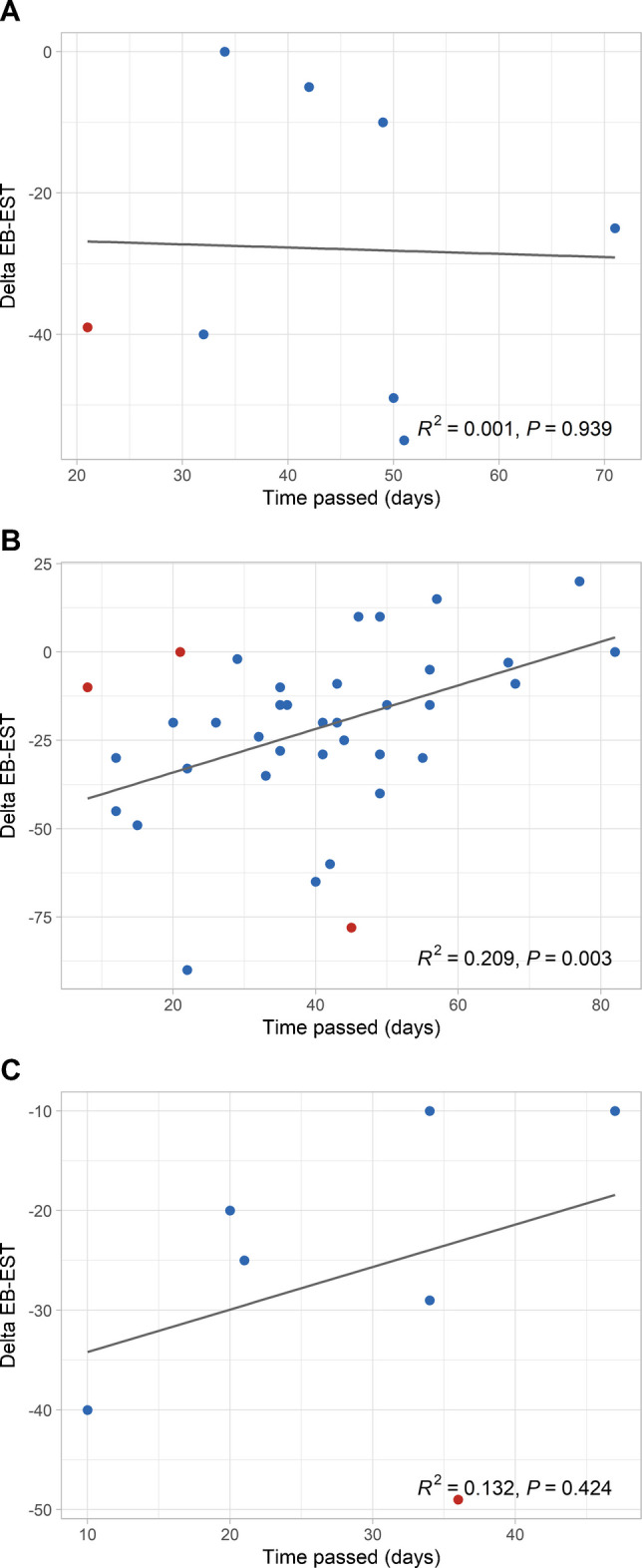
Scatter plot (each point represents an observation) with the fitted trend (line) and model fit (R-squared) illustrating the relationship between change (delta) in the value of Ki67 labelling index and days passed between the last cycle of systemic chemotherapy and surgery for eye-balling-based estimation (EB-EST) according to RCB tumour regression grading scores (subgroup analysis) such as **A** RCB-I, **B** RCB-II, and **C** RCB-III. Statistical outliers are indicated in red. Footnote: RCB, residual cancer burden; RCB-I, RCB score greater than 0 and less than or equal to 1.36; RCB-II, RCB score greater than 1.36 and less than or equal to 3.28; RCB-III, RCB score greater than 3.28

Subgroup analysis based on RCB regression grade scores indicated discordant trendlines between RCB-I versus RCB-II and RCB-III categories, with the former indicating a downward trend and the latter two having an upward trend, meaning a decrease in mitotic rate reduction with time after NACT. RCB-I and RCB-III categories contained a suboptimal number of cases (*n* = 8 and *n* = 7, respectively), which is a limitation of our study. The best goodness of fit was achieved in the RCB-II group (*p* = 0.003, *R*^2^ = 0.209), which also contained the highest number of cases (*n* = 39). The resulting linear regression models with their corresponding *R*^2^ values are presented in Fig. 4.

**Fig. 5:**
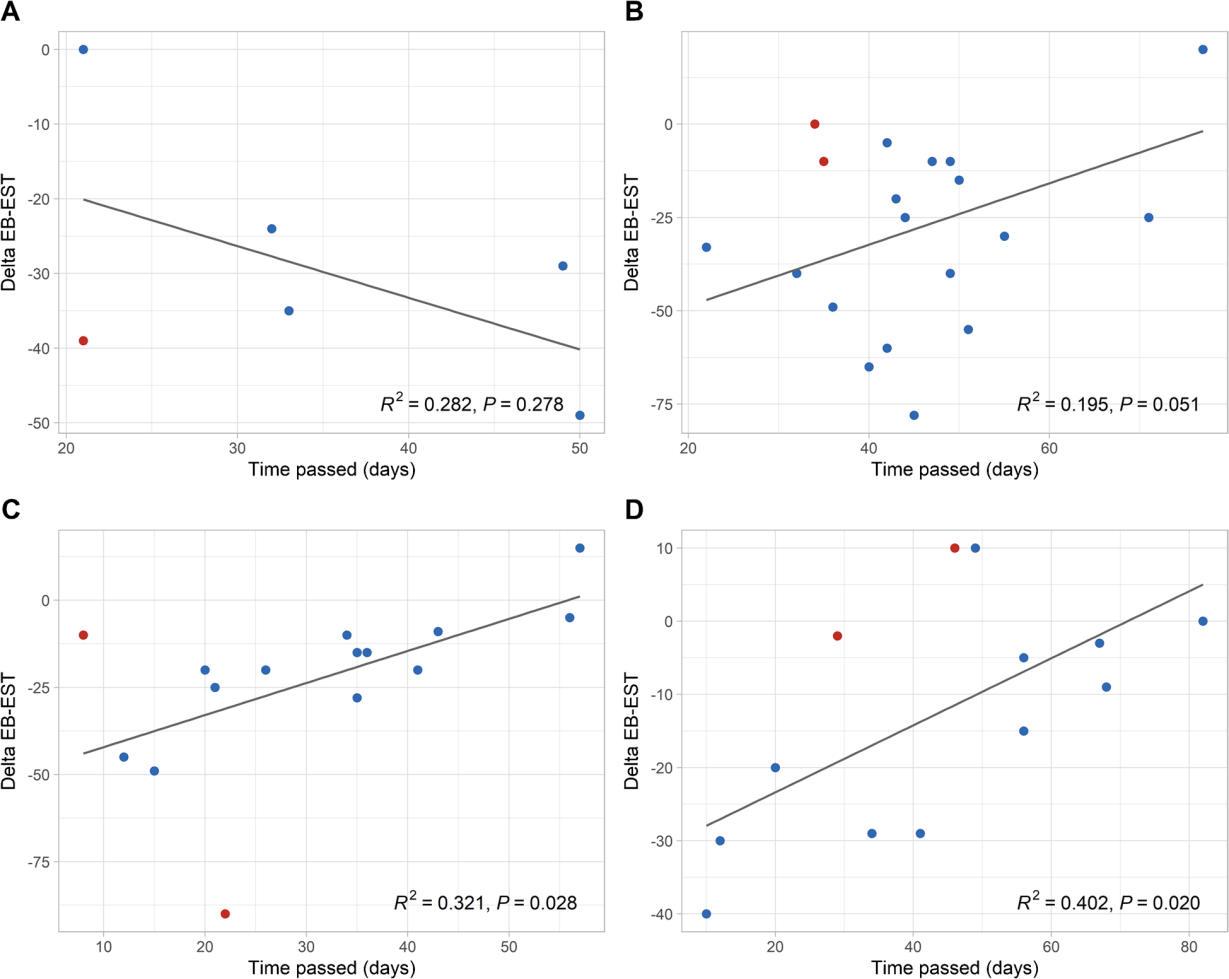
Scatter plot (each point represents an observation) with the fitted trend (line) and model fit (R-squared) illustrating the relationship between change (delta) in the value of Ki67 labelling index and days passed between the last cycle of systemic chemotherapy and surgery for eye-balling based estimation (EB-EST) according to EWGBSP tumour regression scores (subgroup analysis) such as **A**: TR2a, **B**: TR2b, **C**: TR2c and **D**: TR3. Statistical outliers are indicated in red EWGBSP: European Working Group for Breast Cancer Screening, TR2a: < 10% residual cancer, TR2b: 10-50% residual cancer, TR2c: > 50% residual cancer but with signs of regression, TR3: no signs of regression

When analysing the cases according to EWGBSP regression grade categories, the linear regression trendline of TR2a tumours had a downward slope (*p* = 0.278, *R*^2^ = 0.2819), indicating a sustained effect of chemotherapy, while TR2b (*p* = 0.051, *R*^2^ = 0.1951), TR2c (*p* = 0.028, *R*^2^ = 0.3209) and TR3 (*p* = 0.02, *R*^2^ = 0.4019) groups demonstrated an opposite, upward trend in the change of Ki67 over time (Fig. 5).

Regarding the Ki67 LI values in core biopsies, the Bland–Altman (BA) analysis assessing the agreement between the EB-EST and IR, and the EB-EST and GRID (in this order, pairwise) estimation methods showed a mean difference (bias) of 9.3 (95% CI: 4.6 to 14.0; *p* < 0.001) and −4.0 (95% CI: −7.3 to −0.6; *p* = 0.02), with limits of agreement ranging from −24.6 to + 43.2 and −28.1 to + 20.2, respectively (Supplementary Fig. 2). However, no significant pattern (that is, trend) in the differences was observed across the range of the methods pairwise (*p* = 0.874 and *p* = 0.112, respectively).

As for the values in excision specimens, the BA analysis demonstrated an average difference of 9.8 (95% CI: 5.1 to 14.6; *p* < 0.001) and −0.9 (95% CI: −3.0 to + 1.2; *p* = 0.4) between EB-EST and IR, and EB-EST and GRID, respectively. The range for limits of agreement was −24.2 to + 43.9 and −15.9 to + 14.1, respectively (Supplementary Fig. 3). Additionally, significant increasing trends in the differences were detected across the range of methods (*p* < 0.001 and *p* = 0.01, respectively). Specifically, the difference between the estimation methods increases with the magnitude of the Ki67 LI value—that is, EB-EST tends to produce higher values than IR/GRID as values increase (Supplementary Fig. 4).

## Discussion

In recent years, the significance of NACT has been increasing, as it allows for a more conservative surgical approach by down-sizing, as well as monitoring the tumour response to the chemotherapeutic agents and eliminating micro-metastases in high-risk early or locally advanced breast cancers [6]. Pathological complete response (pCR) after NACT has been the focus of multiple studies and has proven to be a good predictor of survival [23]. It has also been noted that pCR is only achieved in a minority of cases after NACT, approximately 15–20%; thus, it remains of great importance to further study possible predictive factors and prognosticators in residual disease [24].

The effectiveness of NACT is highly dependent on the biological behaviour of breast cancer, supported by the fact that it is mostly given to patients with poorly differentiated (G3), highly proliferative tumours, because the proliferation-blocking effects of NACT may be more beneficial in these cases. In our previous work, we were able to reproduce and statistically describe the changes observed in histological grade and its component scores for tubule formation, nuclear pleomorphism, and mitotic rate, which have been reported by other authors as well [25]. Specifically, if there was a change from CNB to EXC, the grade tended to decrease rather than increase as a result of mitotic rate reduction in the group treated with NACT [26]. Being able to monitor the response of a tumour to this proliferation-blocking effect of NACT might help identify patients who might derive less benefit from the given therapies.

Dynamic, biomarker-based adjustment therapy regimens may offer a way to separate the therapeutic pathways of well-responders from non-responders to reach the best-personalised outcome, avoiding under- or overtreatment. In hormone receptor (HR)-positive, HER2-negative intermediate risk tumours, this question has been assessed by the WSG-ADAPT-HR+/HER2- sub-trial, based on Ki67 response. Treatment was tailored based on a second, interim biopsy sample taken after 3 weeks of induction endocrine therapy and its Ki67 LI value. Patients meeting the threshold ≤ 10% Ki67 LI continued to receive endocrine therapy only, while those with Ki67 LI > 10% also received chemotherapy. The trial concluded that Ki67 from interim biopsies was valuable to assess response to therapy. Similarly, the POETIC trial concluded that Ki67 was useful for the differentiation of well-responder and non-responder groups, yKi67 LI ≤ 10% in EXC specimens from surgery 2 weeks after randomization was prognostic, and adjuvant endocrine therapy alone was sufficient for responders, whereas non-responders might benefit from further adjuvant treatments [27–30].

Adjusting NACT regimens based on tumour proliferation change has been studied by Mukai et al., who monitored the effects of NACT by assessing Ki67 from interim biopsy specimens in HER2-positive tumours and divided their patients into Ki67 responder and non-responder groups. They concluded that switching the original weekly paclitaxel and trastuzumab therapy regimen to epirubicin combined with cyclophosphamide and trastuzumab produced worse pCR rates in non-responders than the pCR rates of patients maintained on the original NACT [31]. We are not aware of other studies investigating the adjustment of NACT according to Ki67 response; therefore, further studies of possible therapeutic agents might be needed to reach a better conclusion on Ki67 adjustment of treatment in this subset of patients.

According to the 2015 St. Gallen consensus conference, the main IHC surrogate subtypes of breast cancer are triple-negative breast cancer (TNBC), HR+HER2+, HR-HER2+, and the HR+HER2- groups, the latter consisting of luminal A-like and luminal B-like tumours, among which Ki67 LI is an important differentiating factor [32]. While there is no consensus on the cut-off value between low and high Ki67, the most commonly observed values were between 15 and 30%, the latter being the threshold for high proliferation currently suggested by the International Ki67 in Breast Cancer Working Group and the latest St Gallen Consensus Conference dealing with this issue, while 20% had been the cut-off for separating luminal A-like tumours from luminal B-like ones [1, 6, 12, 13, 23, 33–37].

Chen et al. reported a 37.5% discordance rate in Ki67 LI between CNB and post-NACT EXC specimens using a > 20% cut-off to dichotomise tumours as of high or low proliferation. The survival of patients with pre- and posttreatment Ki67 both ≤ 20% was significantly better than any of the concordantly high or discordant low to high or high to low converted cases [23]. Our previous study comparing cases treated with primary surgery or NACT suggests that based on mitotic counts, discordantly scored biopsies tend to underestimate proliferation in general (primary surgery cases), but in NACT cases, proliferation in core biopsies is more often higher than in post-NACT specimens [26], and this result is in keeping with the results of Chen et al. [23]. Furthermore, we also demonstrated that post-NACT histological grade has also prognostic impact [38].

While ΔKi67 was also recognised as prognostic, yKi67 was the main factor predicting early local and distant metastasis risk and survival, with higher yKi67 values corresponding to poorer prognosis, regardless of the extent of the change [5, 11, 13–15]. Many authors seem to agree on the prognostic value of yKi67 LI [5, 13, 39, 40].

A comprehensive meta-analysis was done by Li et al., examining preoperative and postoperative Ki67 as well as ΔKi67 from biopsy to excision in terms of OS and disease-free survival (DFS). Their study concluded that all three of these markers are independent prognostic factors of both OS and DFS, this finding being backed by other studies since then [12, 24, 41]. Furthermore, yKi67 proved to be a stronger predictor of DFS than of OS [5, 34, 35].

We may conclude from previous studies that Ki67 LI assessed both from CNB and EXC specimens carries important information and has an impact on prognosis. Not only the starting and end values (i.e., CNB and EXC specimen values) but also the degree of change between the two can alter the expected prognosis, and yet, the temporality and trend of this change over time between the last cycle of chemotherapy and surgery remain to be less investigated. Our study aimed to fill this gap in knowledge.

Our cumulative data may suggest that the drop in proliferation after NACT may gradually be lost, and therefore, time from the last chemotherapy administration may also influence the prognostic value of yKi67 and ΔKi67. This suggestion would be more straightforward if all tumours behaved similarly to NACT, which is not the case. When we tried to analyse subsets of tumours according to their molecular subtype using the surrogate molecular classification or the degree of response to NACT, we saw a similar tendency for a drop in yKi67 in nearly all subsets, except for the good prognosis luminal A-like tumours and the tumours with the best response to treatment (RCB class I and EWGBSP TR2a), all underrepresented in the cohort. The data are purely descriptive and seem to reflect a dynamism in the proliferation-reducing effect of NACT with time from termination in most breast cancers, with the potential exception of the luminal A-like tumours (unlikely to receive NACT) or the ones with good response to treatment, where the effect may be more permanent. We used different methods of assessment, including two objective ones based on sampling (IR, GRID) and a thoroughly carried out but subjective one (EB-EST), and all showed similar trends (Fig. 2); we have finally selected EB-EST based on the best goodness-of-fit and its widespread use in clinical practice. The absolute values of Ki67 differed by method, and probably artificial intelligence-based quantification would have been the most robust support for substantiating our findings, but due to the current unavailability of this technology in our hands, we sought to demonstrate the similarities in the results using different methods of assessment.

As a limitation, the relatively small sample size of this study interferes with the interpretation of the results in the smallest subsets. Eliminating outliers could have improved the goodness-of-fit of the regression models, but low case numbers have hampered this.

## Conclusions

In conclusion, our findings reinforce a drop in proliferation following NACT in most breast carcinomas evaluated, and suggest that this drop may decrease with increasing time between the last cycle of NACT and surgery. This dynamism was seen in more aggressive molecular subtypes and tumours with lesser response to treatment, and might be missing from the rare luminal A-like tumours or carcinomas with better response to treatment, though conclusions for these latter subgroups were limited by low case numbers.

## Supplementary Information

ESM 1 (ZIP 11.7 MB)

ESM 2 (TIFF 302 KB)

ESM 3 (TIFF 296 KB)

ESM 4 (TIFF 318 KB)

ESM 5 (DOCX 27.1 KB)

ESM 6 (DOCX 19.5 KB)

## Data Availability

Anonymised data collected and analysed are stored in an Excel file with Hungarian labels and are available from the corresponding author upon reasonable request.
