# Supplementary material for: Temporality of change in Ki67 labelling following neoadjuvant systemic chemotherapy of breast cancer—the common drop often subsides with time
Source: Virchows Arch. 2025 Nov 13;489(2):297–306. doi: 10.1007/s00428-025-04263-7 (PMC13477442; doi:10.1007/s00428-025-04263-7)
Supplement: Supplementary file 1 [file 428_2025_4263_MOESM5_ESM.docx]

Supplementary Table 1.

Clinical data of the patients and tumours analysed.

| Age (mean; median; range) |  | 57.9; 58; 32-78 |
| --- | --- | --- |
| ER status |  |  |
|  | Positive | 32 |
|  | Negative | 22 |
| PR status |  |  |
|  | Positive | 29 |
|  | Negative | 25 |
| HER2 status |  |  |
|  | Positive | 12 |
|  | Negative | 42 |
| Grade |  |  |
|  | I | 2 |
|  | II | 18 |
|  | III | 34 |
| yGrade |  |  |
|  | I | 6 |
|  | II | 33 |
|  | III | 15 |
| cTNM |  |  |
|  | cT1b | 1 |
|  | cT1c | 16 |
|  | cT2 | 30 |
|  | cT3 | 6 |
|  | cT4 | 1 |
|  |  |  |
|  | cN0 | 31 |
|  | cN+ | 23 |
| ypTNM |  |  |
|  | ypT1a | 3 |
|  | ypT1b | 13 |
|  | ypT1c | 25 |
|  | ypT2 | 10 |
|  | ypT3 | 1 |
|  | ypT4 | 2 |
|  | ypN0 | 30 |
|  | ypN1 | 18 |
|  | ypN2 | 5 |
|  | ypN3 | 1 |
| NACT regimens |  |  |
| LumA-like | EC+Taxane | 3 |
|  | Capecitabine | 1 |
| LumB-like | EC+Taxane | 22 |
| TN | EC+Taxane | 15 |
|  | EC | 1 |
| HER2 positive | EC+Taxane | 2 |
|  | EC+Taxane+Trastuzumab | 3 |
|  | EC+Taxane+Trastuzumab+Pertuzumab | 3 |
|  | EC+Taxane+Carboplatin+Trastuzumab | 2 |
|  | EC+Taxane+Carboplatin+Trastuzumab+Pertuzumab | 1 |
|  | EC+Taxane+5-FU+Trastuzumab | 1 |
