# Supplementary material for: Temporality of change in Ki67 labelling following neoadjuvant systemic chemotherapy of breast cancer—the common drop often subsides with time
Source: Virchows Arch. 2025 Nov 13;489(2):297–306. doi: 10.1007/s00428-025-04263-7 (PMC13477442; doi:10.1007/s00428-025-04263-7)
Supplement: Supplementary file 2 [file 428_2025_4263_MOESM6_ESM.docx]

Supplementary table 2: Median decrease in Ki67 LI by surrogate molecular type and time from last cycle of chemotherapy

|  | Time from last cycle of NACT | | |  |  |
| --- | --- | --- | --- | --- | --- |
| Surrogate molecular type | 8-20 days | 21-40 days | 41-60 days | 61-82 days | |
| Luminal B-like (HER2-) | 49 (1) | 28.5; 20 (12) | 27; 12 (8) | 25 (1) | |
| TNBC | 32.5; 20-45 (2) | 33; 0-65 (3) | 17.5; 50 (8) | -20; -20-0 (3) | |
| HER2+ | 25; 20 (4) | 15; 10-20 (2) | 5; 27 (5) | 3 (1) | |
| Luminal A-like | - | 1; 0-2 (2) | 10 (1) | 9 (1) | |

Data are presented as: median; interquartile range (n), where n is the number of cases in the given category; this is modified if the number of cases is limited, i.e. 1 (no range is given) and 2 or 3 (only the minimum and maximum value range is given).
