# Supplementary material for: Temporality of change in Ki67 labelling following neoadjuvant systemic chemotherapy of breast cancer—the common drop often subsides with time
Source: Virchows Arch. 2025 Nov 13;489(2):297–306. doi: 10.1007/s00428-025-04263-7 (PMC13477442; doi:10.1007/s00428-025-04263-7)

Supplementary figure 1

- Pairs of core needle biopsies (CNB; left) and excision specimens (EXC; right) with tumour regression categories (TR) and residual cancer burden (RCB) values and categories as detailed in the methods. The examples are from digitized diagnostic haematoxylin and eosin (HE) stained slides; magnifications (x…) are derived from digitized slides (SlideViewer, 3DHistech Budapest).
- For information, the eyeballing estimates (EB-EST) of Ki67 labelling are given in parentheses for both the pre-treatment (CNB) and post-treatment (EXC) tumours.

A

Example 1: Case showing no regression: TR3, RCB= 1.551 (II)

CNB (EB-EST: 70%) EXC (EB-EST: 80%)

(both HE x1)

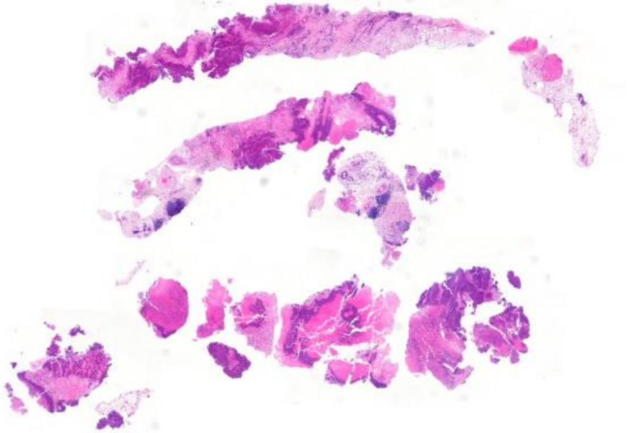

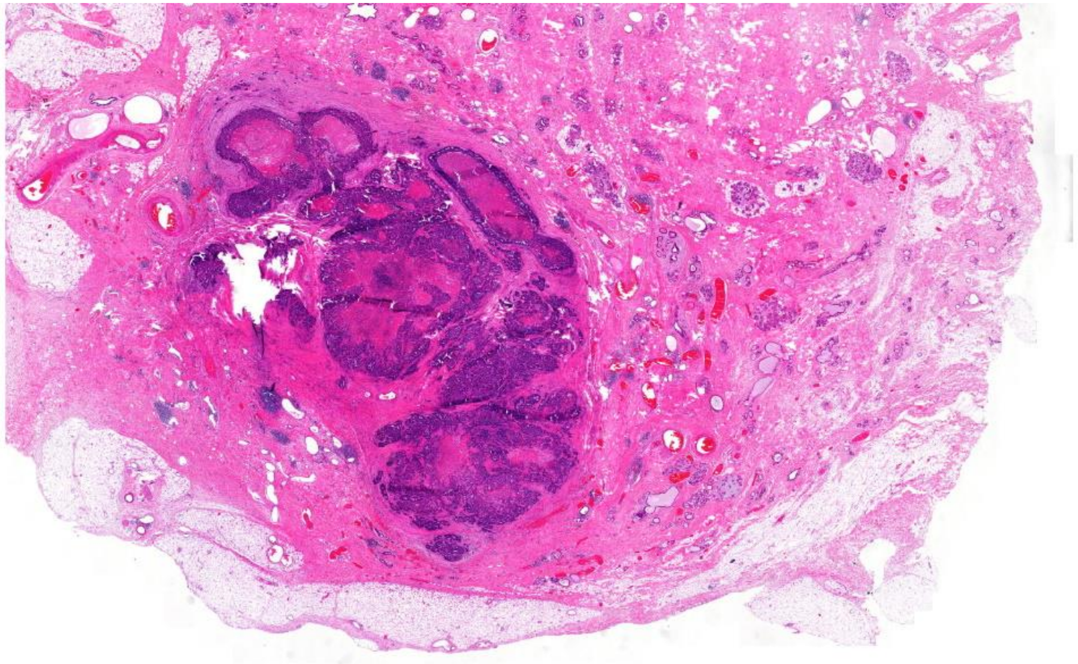

Triple-negative, ER- PR- HER2 0; cT2 cN0 → ypT1c ypN0(sn)

B

Example 1: Case showing no regression: TR3, RCB= 1.551 (II)

CNB (EB-EST: 70%) EXC (EB-EST: 80%)

(both HE x5)

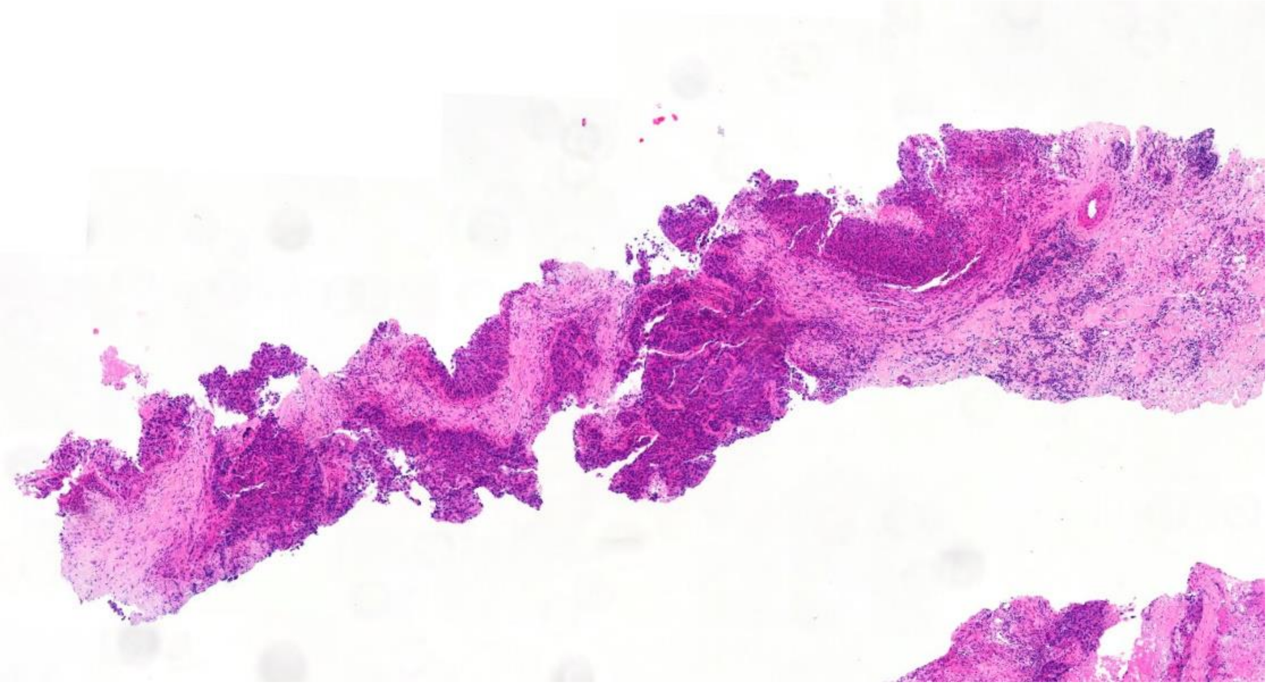

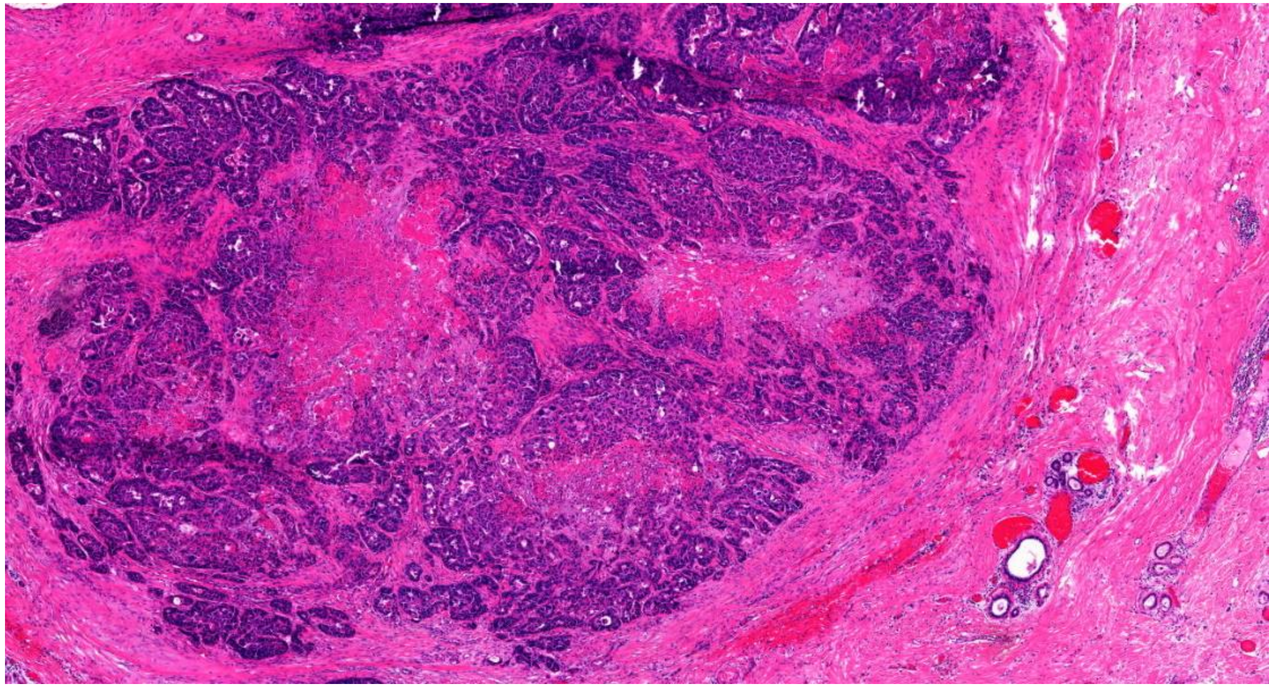

C

Example 1: Case showing no regression: TR3, RCB= 1.551 (II)

CNB (EB-EST: 70%) EXC (EB-EST: 80%)

(both HE x20)

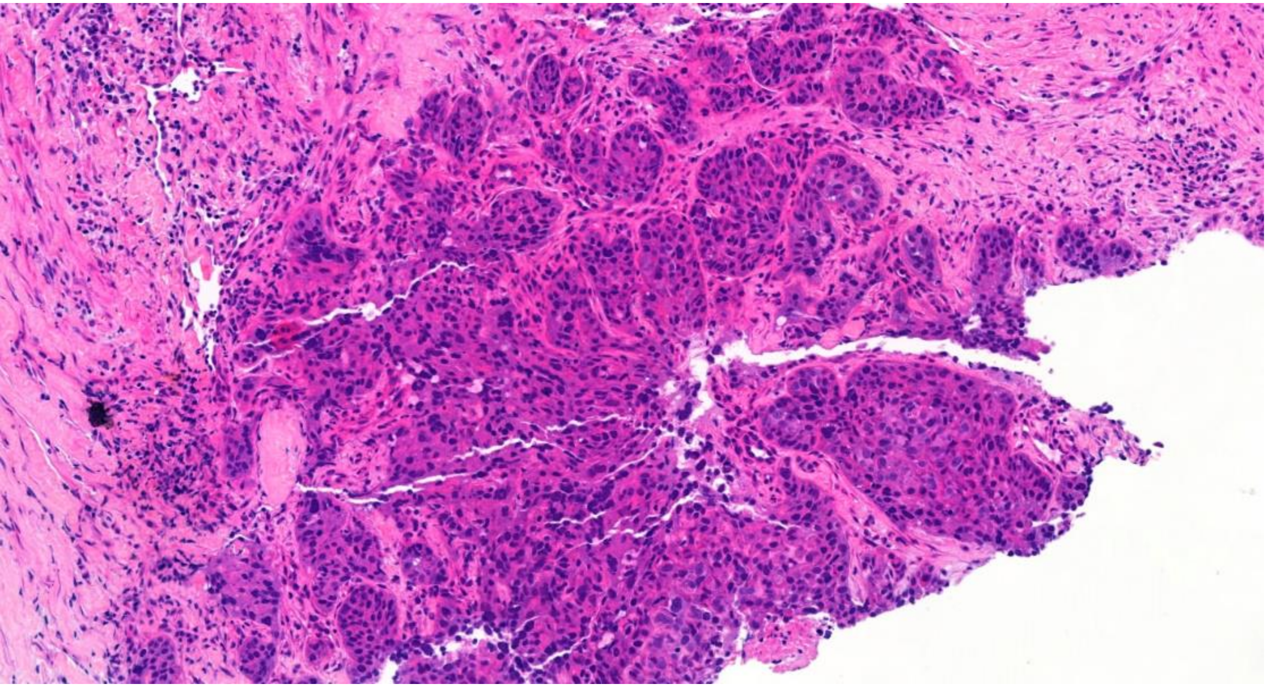

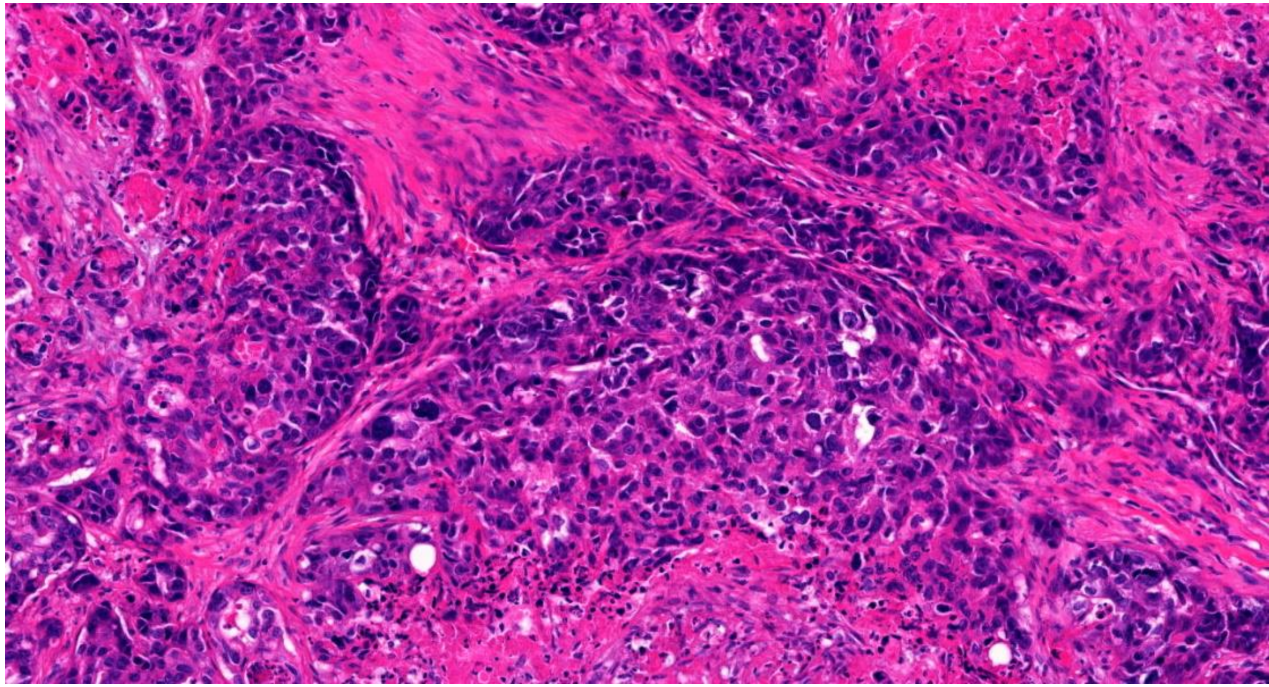

D

Example 1: Case showing no regression: TR3, RCB= 1.551 (II)

CNB (EB-EST: 70%) EXC (EB-EST: 80%)

(both HE x40)

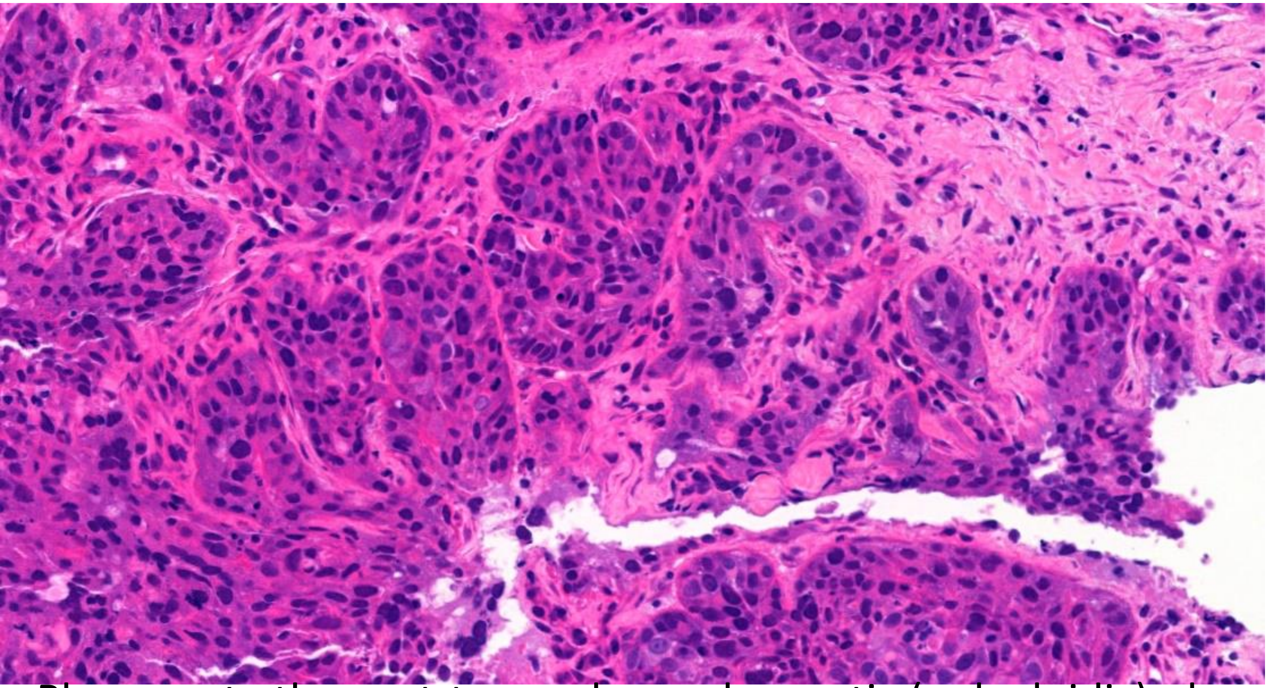

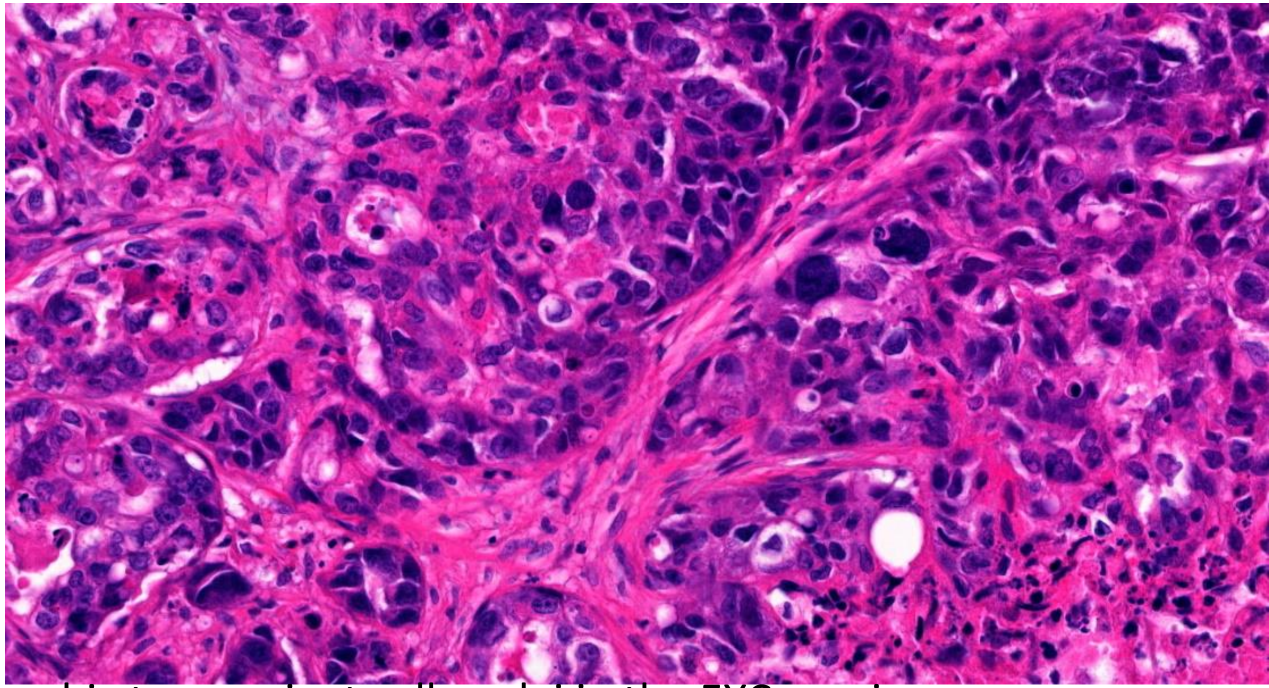

Please note the post-taxane hyperchromatic (polyploidic) pleomorphic tumor giant cell nuclei in the EXC specimen

(e.g.: Ferenczi Á, Cserni G. Changes in breast cancer grade from biopsy to excision following surgery or primary chemotherapy. Pathologica 2024;116:22-31. doi: 10.32074/1591-951X-958

Zombori T, Cserni G. Patterns of regression in breast cancer after primary systemic treatment. *Pathol Oncol Res.* 2019;25:1153-1161. doi: 10.1007/s12253-018-0557-7

Valent A, Penault-Llorca F, Cayre A. Change in HER2 (ERBB2) gene status after taxane-based chemotherapy for breast cancer: polyploidization can lead to diagnostic pitfalls with potential impact for clinical management. *Cancer Genet.* 2013;206:37-41. doi: 10.1016/j.cancergen.2012.12.001

E

Example 2: Case showing minor regression: TR2c, RCB= 3.302 (III)

CNB (EB-EST: 30%) EXC (EB-EST: 5%)

(both HE x1)

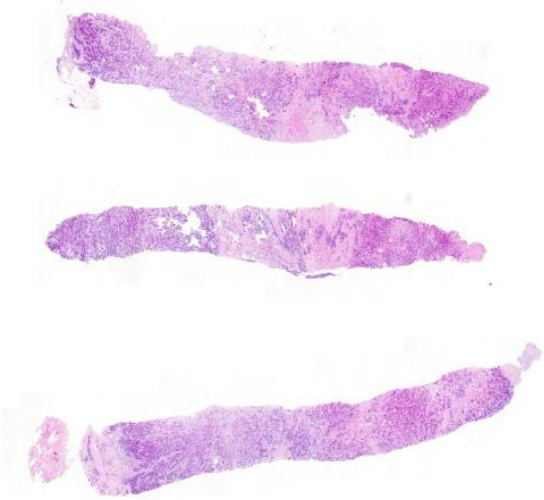

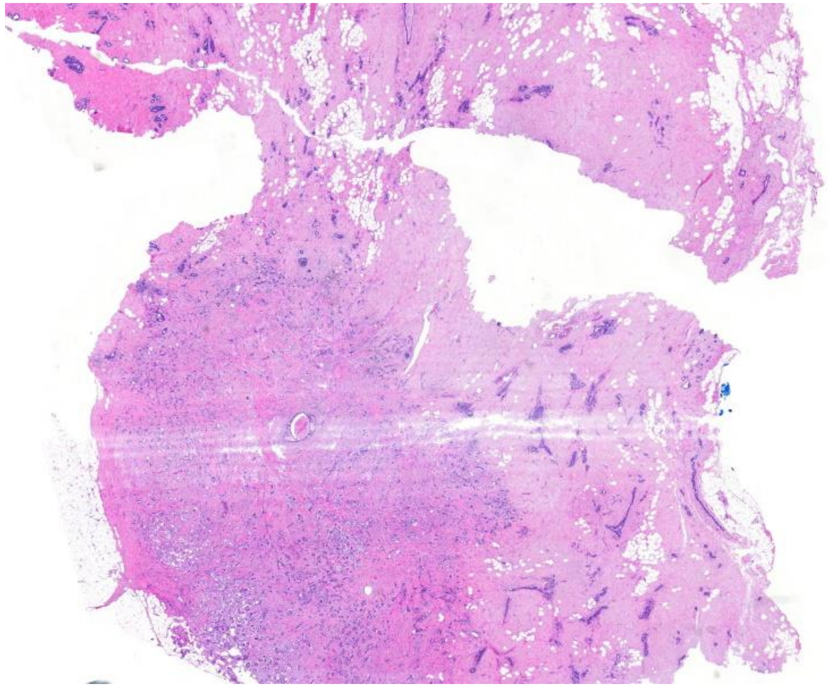

Luminal B-like, ER+ PR+ HER2 0; cT2 cN1(f) → ypT1c ypN2a

F

Example 2: Case showing minor regression: TR2c, RCB= 3.302 (III)

CNB (EB-EST: 30%) EXC (EB-EST: 5%)

(both HE x5)

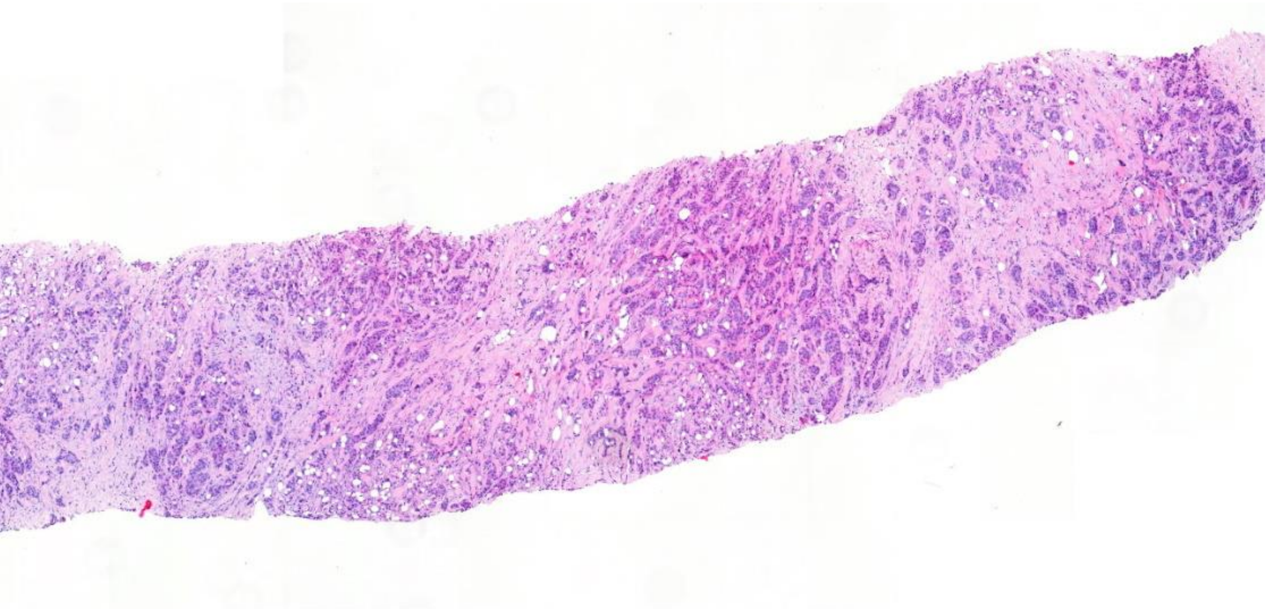

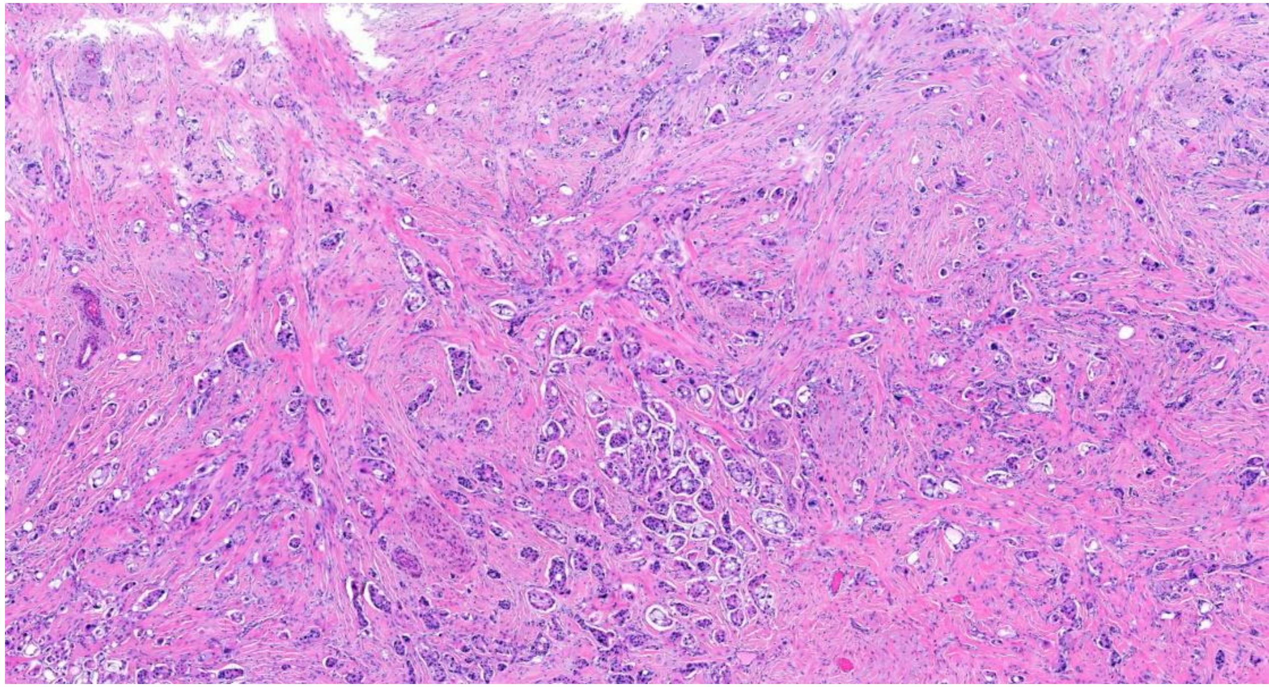

G

Example 2: Case showing minor regression: TR2c, RCB= 3.302 (III)

CNB (EB-EST: 30%) EXC (EB-EST: 5%)

(both HE x20)

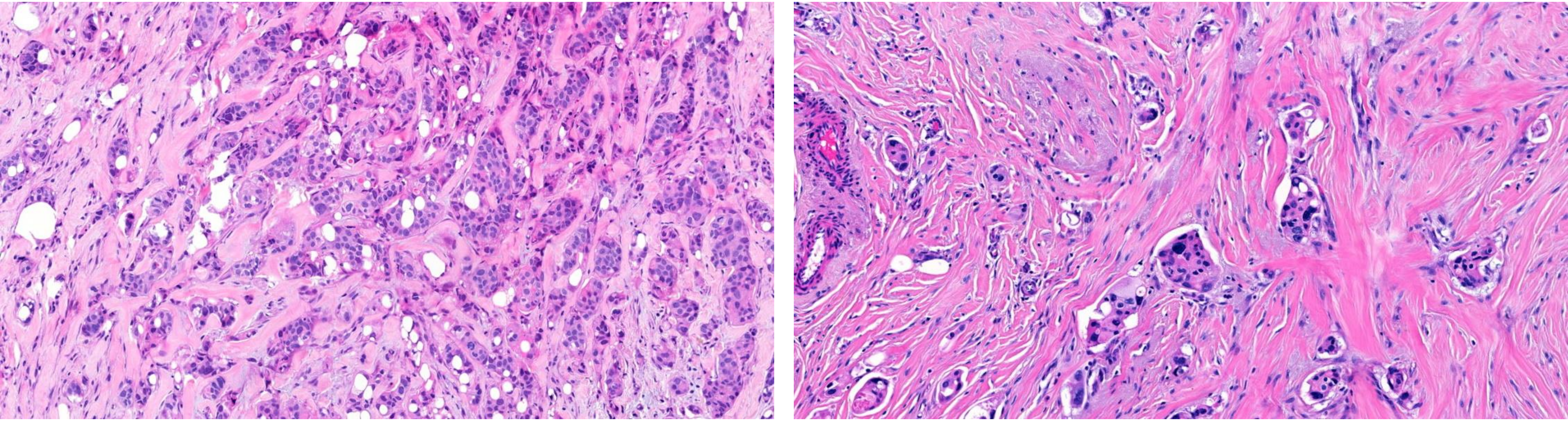

H

Example 2: Case showing minor regression: TR2c, RCB= 3.302 (III)

CNB (EB-EST: 30%) EXC (EB-EST: 5%)

(both HE x40)

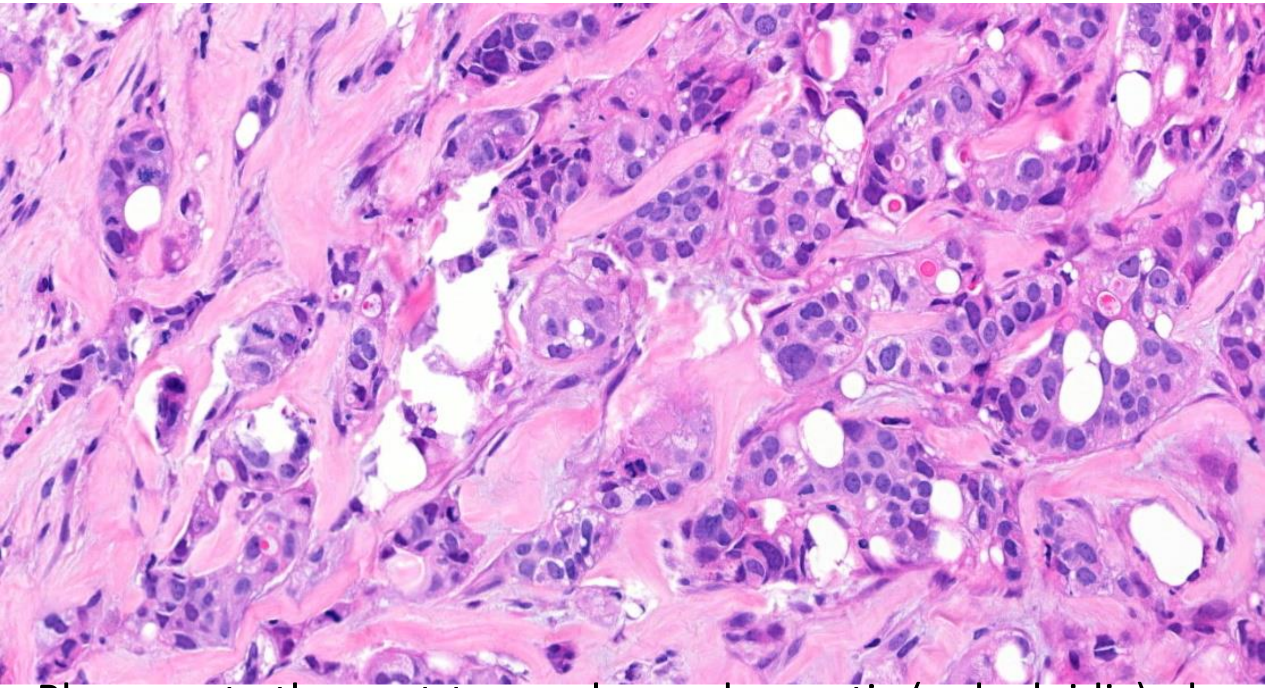

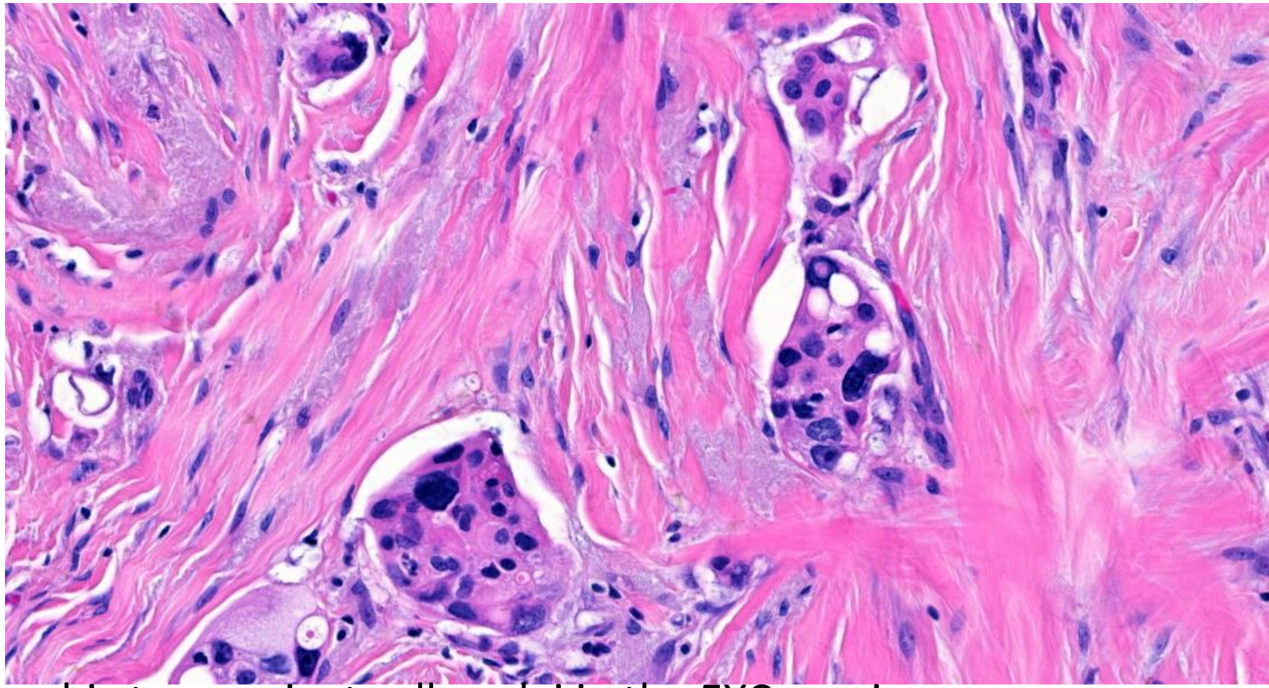

Please note the post-taxane hyperchromatic (polyploidic) pleomorphic tumor giant cell nuclei in the EXC specimen

(e.g.: Ferenczi Á, Cserni G. Changes in breast cancer grade from biopsy to excision following surgery or primary chemotherapy. Pathologica 2024;116:22-31. doi: 10.32074/1591-951X-958
Zombori T, Cserni G. Patterns of regression in breast cancer after primary systemic treatment. *Pathol Oncol Res.* 2019;25:1153-1161. doi: 10.1007/s12253-018-0557-7
Valent A, Penault-Llorca F, Cayre A. Change in HER2 (ERBB2) gene status after taxane-based chemotherapy for breast cancer: polyploidization can lead to diagnostic pitfalls with potential impact for clinical management. *Cancer Genet.* 2013;206:37-41. doi: 10.1016/j.cancergen.2012.12.001

Example 3: Case showing significant regression: TR2a, RCB= 1.132 (I)

CNB (EB-EST: 50%) EXC (EB-EST: 1%)

(both HE x1)

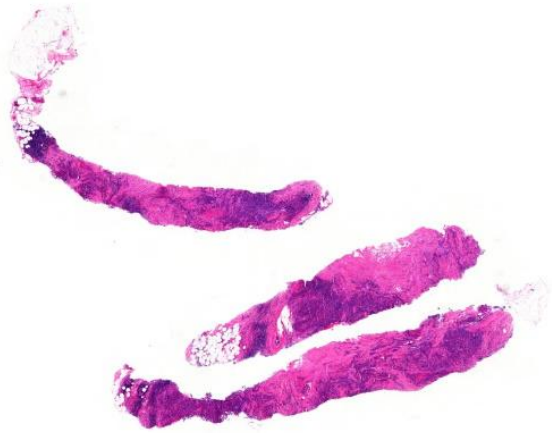

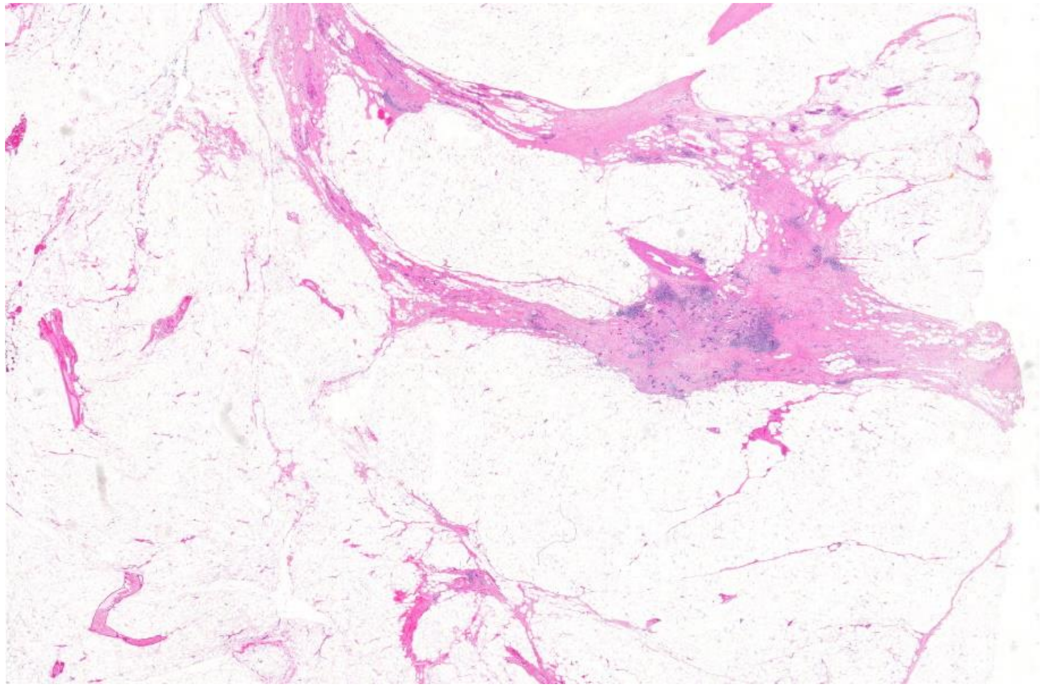

Luminal B-like, ER+ PR+ HER2 0; cT1b cN1(f) → ypT1a ypN0

Example 3: Case showing significant regression: TR2a, RCB= 1.132 (I)

CNB (EB-EST: 50%) EXC (EB-EST: 1%)

(both HE x5)

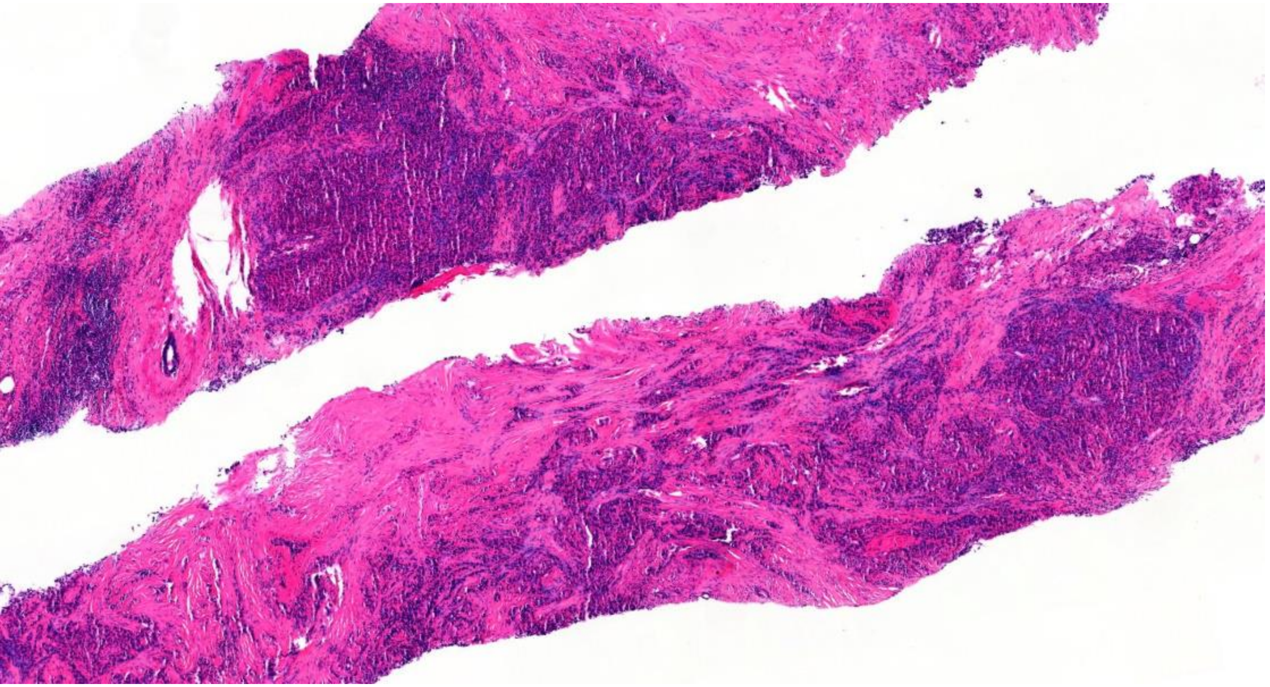

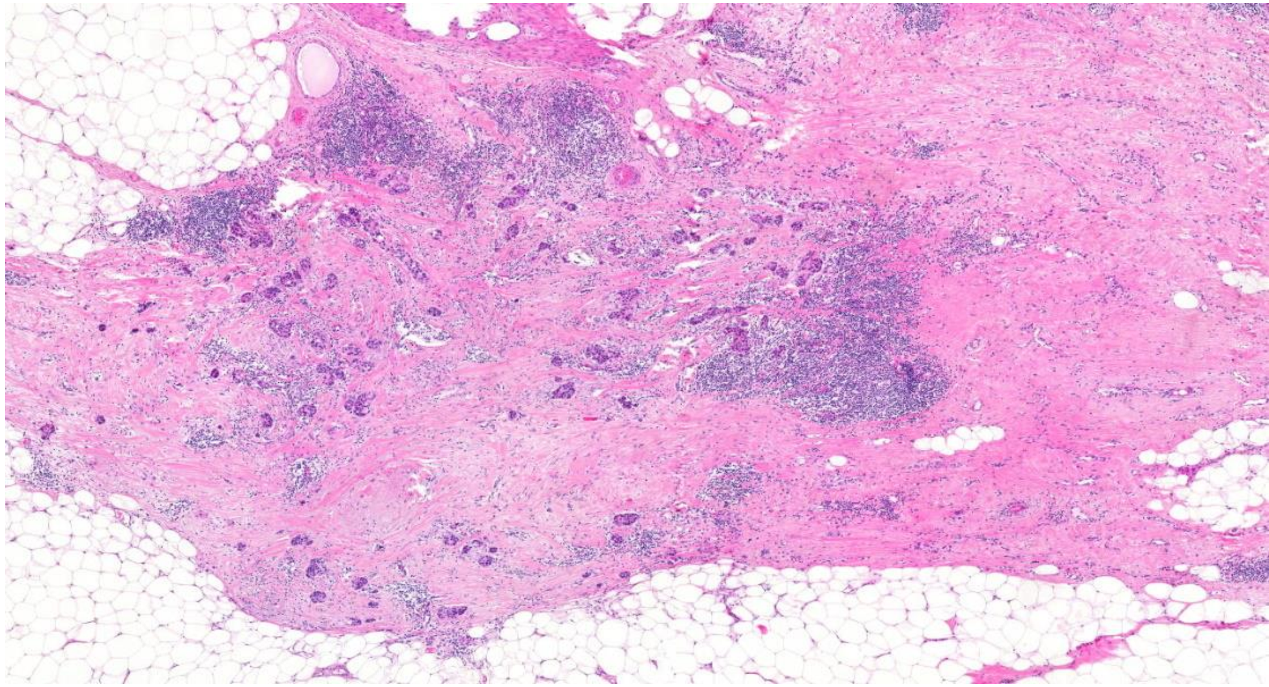

K

Example 3: Case showing significant regression: TR2a, RCB= 1.132 (I)

CNB (EB-EST: 50%) EXC (EB-EST: 1%)

(both HE x20)

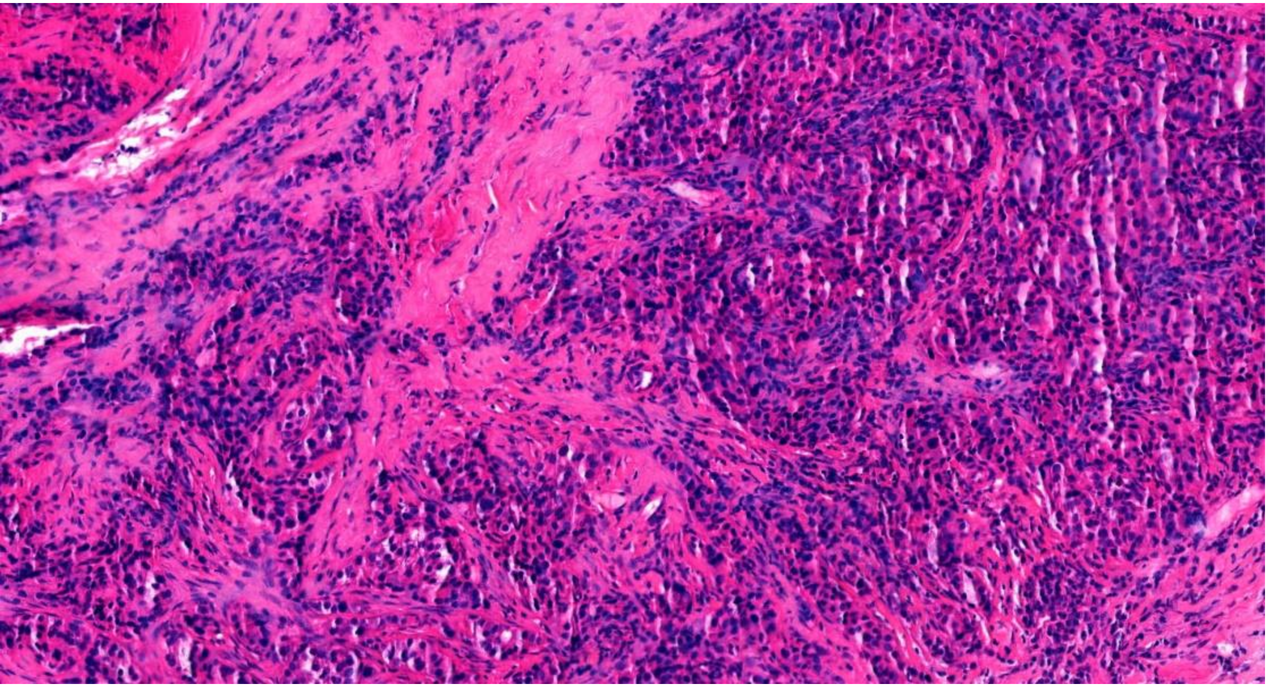

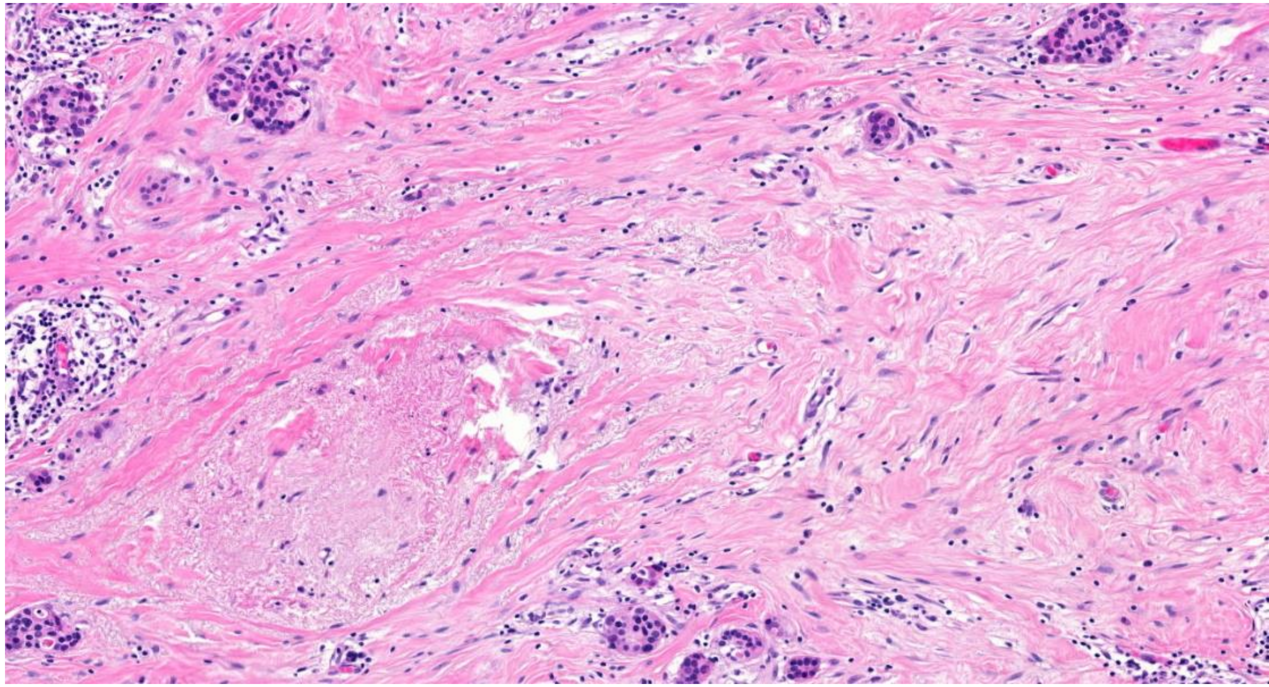

L

Example 3: Case showing significant regression: TR2a, RCB= 1.132 (I)

CNB (EB-EST: 50%) EXC (EB-EST: 1%)

(both HE x40)

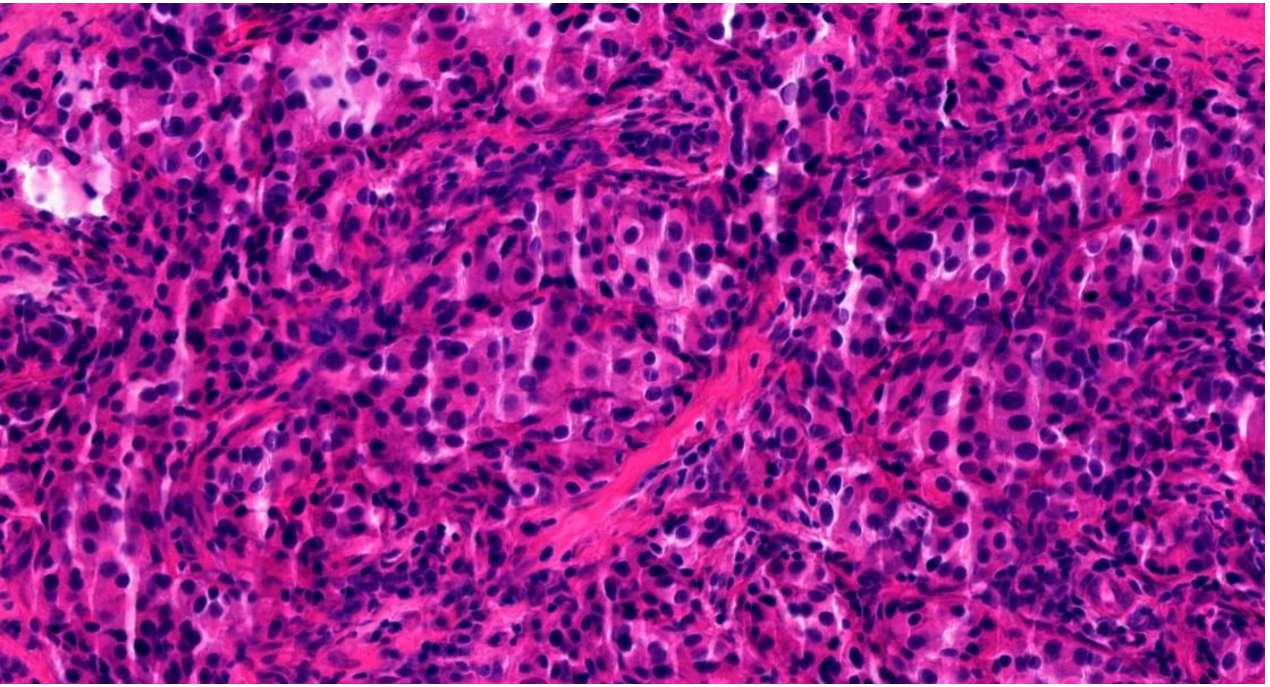

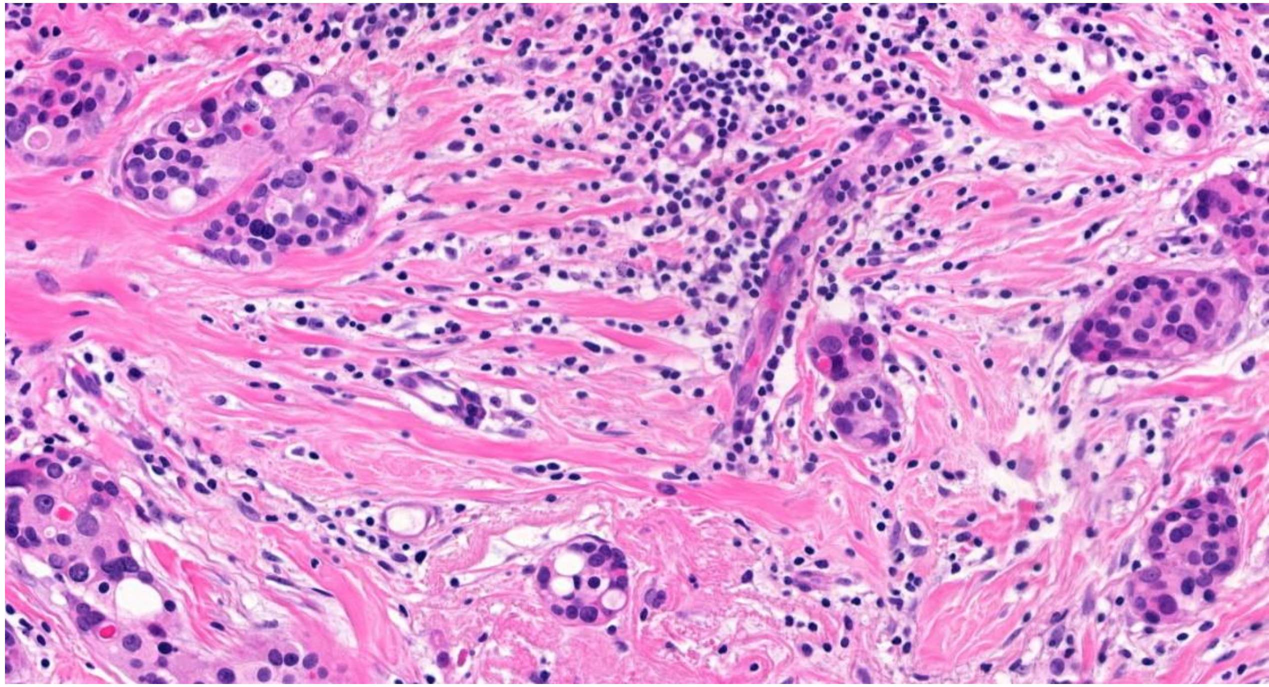

Supplement: Supplementary file 3 [file 428_2025_4263_MOESM1_ESM.zip › Supplementary figure 1.pdf]
